# Implementation of a Community-Based Public Model for the Prevention and Control of Communicable Diseases in Migrant Communities in Catalonia

**DOI:** 10.3390/tropicalmed8090446

**Published:** 2023-09-14

**Authors:** Jordi Gómez i Prat, Helena Martínez Alguacil, Sandra Pequeño Saco, Hakima Ouaarab Essadek, Jordi Montero i Garcia, Oriol Catasús i Llena, Jacobo Mendioroz Peña

**Affiliations:** 1 Public Health and Community Team (eSPiC), Unit of Tropical Medicine and International Health Drassanes-Vall d’Hebron (UTMIHD-VH), PROSICS, 08001 Barcelona, Spain; jordi.gomez@vallhebron.cat (J.G.i.P.);; 2 Agència de Salut Pública de Catalunya, Departament de Salut, Generalitat de Catalunya, 08005 Barcelona, Spain; 3 inLab FIB, Universitat Politècnica de Catalunya, 08034 Barcelona, Spain

**Keywords:** community-based strategy, communicable diseases, community health workers, vulnerable communities

## Abstract

In high-income countries, migrant populations have a greater epidemiological vulnerability: increased exposure to infectious diseases, difficulties in diagnosis, case follow-up and contact tracing, and obstacles following preventive measures related to cultural and administrative barriers. This study aims to describe the implementation of a community-based program to address these challenges. The target population is the migrant native population from North Africa, South Asia, Sub-Saharan Africa, Eastern Europe, and Latin America resident in Catalonia during 2023. Implementation phases include the identification of the perceived needs, search, recruitment and capacity building of 16 community health workers, and the development of a computer software. From January to June 2023, 117 community-based interventions have been implemented, reaching 677 people: 73 community case and contacts management interventions, 17 community in-situ screenings (reaching 247 people) and 27 culturally adapted health awareness and education actions (reaching 358 people). The program addresses the following infectious diseases: tuberculosis, Chagas disease, hepatitis C, typhoid, scabies, hepatitis B, mumps and tinea capitis. The implementation of a community-based model may be key to improving surveillance communicable diseases, promoting an equitable and comprehensive epidemiological surveillance system.

## 1. Introduction

Infectious diseases remain a public health challenge for all countries. Sustainable Development Goals target 3.3 calls for an end to the epidemics of HIV, tuberculosis (TB), malaria and neglected tropical diseases (NTDs) by 2030, and for efforts to combat viral hepatitis [1].

Migrant communities in high income countries represent a challenge for the achievement of this target [2,3]. The foreign population in Catalonia in 2022 represented 16.3% of the total inhabitants [4]. Migration may be a determinant of health that interacts with other factors including precarious housing and working conditions, cultural, linguistic and legal barriers, and even some forms of exclusion, fear and stigma [5]. These factors can lead to an increased exposure to infectious diseases and, in general, poorer health.

Immigrants in a situation of social vulnerability tend to have greater epidemiological vulnerability, understood as a contagiom risk due to greater exposure to infectious diseases, delayed diagnosis and contact tracing, and greater difficulty in following preventive and therapeutic measures [6]. This is due, among other factors, to the limited effectiveness of some current strategies, which represents a public health challenge for the management of infectious diseases and epidemic outbreaks in these communities.

Most research on the access of immigrants to health services has been carried out in United States, Canada and some European countries (United Kingdom and the Netherlands) [7]. It tends to focus on the health status of undocumented immigrants, pointing to their predisposition to have lower life expectancy and higher mortality rates for certain causes than natives of the host country [5,7]. This is probably due to worse living, working and socio-economic conditions, corresponding to the difficulty of health services to approach their cultural, linguistic and economic barriers to [7,8,9,10].

The impact of the SARS-CoV-2 pandemic has exacerbated the health inequities, especially for communities with vulnerabilities related to living conditions and may have contributed to the increase in other notifiable diseases [11].

Since social and economic deficiencies increase vulnerability to health problems and limitations to access and use of health resources, it is advisable to adopt measures for social inclusion and improvement of the living conditions of the most disadvantaged immigrants [12]. In this sense, it is important when studying the immigrant population to consider that it is a very diverse group with respect to culture and ethnic characteristics, historical roots and health-related practices [13].

This scenario raises the need to seek new approaches to communicable disease surveillance, with a special focus on migrant population. In this regard, the implementation of community-based strategies incorporating Community Health Workers (CHW) has bridged the gap between migrant communities and the health system. CHWs play an active role in the community and allow a holistic approach for the control of infectious diseases integrating health promotion with socio-cultural engagement [14].

In this context, the Public Health and Community team (eSPiC) of the Drassanes International Health Unit, Vall d’Hebron (Infectious Diseases Service of the Vall d’Hebron Hospital), has participated with the epidemiological surveillance network to develop a community-oriented program to ameliorate the health of migrant communities in Catalonia. Their intervention has proved to play a major role in the detection and access to diagnosis and treatment of Chagas disease in previous years using community strategies and educational tools already validated with the community [15,16,17,18].

The post-pandemic context justified the need to enhance this model based on community health strategies throughout Catalonia in order to address the prevention and control of communicable diseases affecting migrant communities especially in those who are in a situation of epidemiological and social vulnerability.

The general objective of this study is: (i) to describe the implementation the Model of Community and Public Action (MACIP) in Catalonia; and as specific objectives (ii) to describe the socio-economic characteristics of the program’s participant population; and (iii) to describe preliminary results, basically focused on (a) networking and dynamization of the community network; (b) health promotion and response to community needs; (c) community in-situ screening, for selected diseases according to epidemiologic data; and (d) case management and contact tracing in cases with socio-cultural and vulnerability characteristics.

These approaches were complementary to each other. Once a community need is detected, the later tool and strategy are established jointly by the epidemiological surveillance and promotion services of the Public Health Agency of Catalonia (ASPCAT) and the eSPiC.

## 2. Materials and Methods

### 2.1. Study Design

A descriptive study was conducted, from January 2023 to June 2023 about the implementation and preliminary results of the MACIP project. The MACIP project, it is currently in a preliminary phase (pilot phase) that is expected to last two years (until December 2024), after which a comprehensive evaluation will be carried out to assess the applicability and feasibility of expansion of the project.

### 2.2. Study Population

Target population was defined, as the immigrants in a situation of social vulnerability tend to be in a situation of greater epidemiological vulnerability, understood as a higher epidemiological risk due to greater exposure to infectious diseases, delayed diagnosis and contact tracing, and greater difficulty in following preventive and therapeutic measures. All native to North Africa, South Asia, Sub-Saharan Africa, Eastern Europe and Latin America residing in Catalonia during 2023 who have a communicable disease in a vulnerable situation or who could benefit from a community intervention for prevention of these disease, were included. The Epidemiological Surveillance Services carried out the detection and decision to include the patients, therefore no sample was calculated.

### 2.3. Etical Concern

Ethical approval was not required for this descriptive study. All the subjects who were interviewed gave verbal consent to participate in the in the community strategy carried out. This is part of the conventional protocol of the protocols established in different of the notifiable infectious diseases addressed. All patients’ data were codified and analyzed anonymously. No data containing personal or identifying information from the participants have been published.

### 2.4. Phases of Implementation

#### 2.4.1. Actions Priorization

In order to identify and prioritize needs from both a public health and community perspective, a multidisciplinary core group was formed, including the territorial epidemiological surveillance and health promotion services of the ASPCAT, and a specialized public and community health team working in the field of immigration and international health (eSPiC) jointly with a group of CHWs from different cultures.

A detailed incidence map was used to identify the difficulties and needs of each territory regarding the most prevalent infectious diseases among migrant vulnerable populations. The aim of this approach was to carry out a community diagnosis to identify which specific community actions should be priorized in each territory and thus target the profiles of CHWs to be recruited on later phases.

#### 2.4.2. Search and Recruitment of CHWs

The epidemiological surveillance and health promotion services, together with eSPiC, proposed candidates among professionals that were already working in the local community network, and contacted associations from these communities. 

The required profile aimed to incorporate people from the same community, with knowledge of their own culture, community and language, as well as Catalan and Spanish. They were required to be involved in the community network and be familiar with the different community assets and public services. Previous experience in community health work and the ability to work within multidisciplinary groups were positively assessed.

The selection and recruitment process was done through a public call. Based on the curricular profile and personal interviews, the most relevant profiles were selected.

#### 2.4.3. Capacity Building of CHWs

Next step was to capacitate the CHWs through training sessions on epidemiological control of infectious diseases, health promotion and community health. Each session, delivered by professional experts on their respective field, had a duration of eight hours and were distributed in five modules during two days.

The first module covered the general aspects of the ASPCAT. The second module focused on epidemiological control and addressed tuberculosis, scabies outbreaks and vector-borne diseases. The third module, dedicated to health promotion, covered the topics of community intervention, food and non-cariogenic diet, physical activity and dental hygiene. On the second day, the fourth module covered the topics of community health workers and community mobilization. Last module described the work circuits of the project and the presentation of HSUS (Health Survey System) tool for data collection.

In addition, the project had a continuous training plan to provide CHWs with tools and resources to deal with new situations that may emerge, as well as to strengthen their knowledge and skills.

#### 2.4.4. Implementation of HSUS (Health SUrvey System)

In parallel an IT tool was designed for real-time monitoring and evaluation of the community interventions in collaboration with the Polytechnic University of Catalonia.

This tool was designed to record all the activities related to infectious cases management and contact tracing, to register the results of community screening interventions (phone calls, visits, workshops, etc.) and to collect relevant socio-demographic data of the participants in the community actions.

#### 2.4.5. Community-Based Strategy

Community-based strategies used in this project were based on the “eSPiCTools—ACTUA!” model “https://espictools.cat/ (accessed on 7 July 2023)”, an innovative and dynamic model of Research, Training and Action that generates evidence-based educational tools for health promotion through a process of co-creation between health professionals and the community. These are adapted tools designed to strengthen people’s skills and abilities, and to build a relationship with others integrating bio-psycho-social dimensions.

So far, educational tools have been previously validated with the community for the following infectious diseases: CHD [18], hepatitis [19] and HIV [20]. In addition, community strategies for the following infectious were also validated with the community: tuberculosis [21], hepatitis [22] and CHD [15,16,17].

Educational tools and strategies were adapted to the social and cultural aspects of the different populations, according to methodological guidelines previously defined.

## 3. Results

### 3.1. Needs Identified in the Epidemiological Surveillance of Communicable Diseases in the Migrant Population

In terms of epidemiological needs, communities with the highest burden of infectious diseases and/or the greatest barriers to accessing this population were priorized. In this sense initial actions were taken for tuberculosis, scabies and food-outbreaks control.

Another need was the decentralization of the model ensuring territorial equity and community specific approach. The CHWs teams should reach all the prioritized communities and be able to move throughout the territory.

### 3.2. Selection, Recruitment and Capacity Buidling of CHWs

For the selection process, contact was made with various organizations working with migrant communities to find the best candidates for each territory and community: immigrants organizations, government institutions, town councils and people who had participated in previous training courses for CHWs. The selection incorporated the gender perspective.

**Figure 1:**
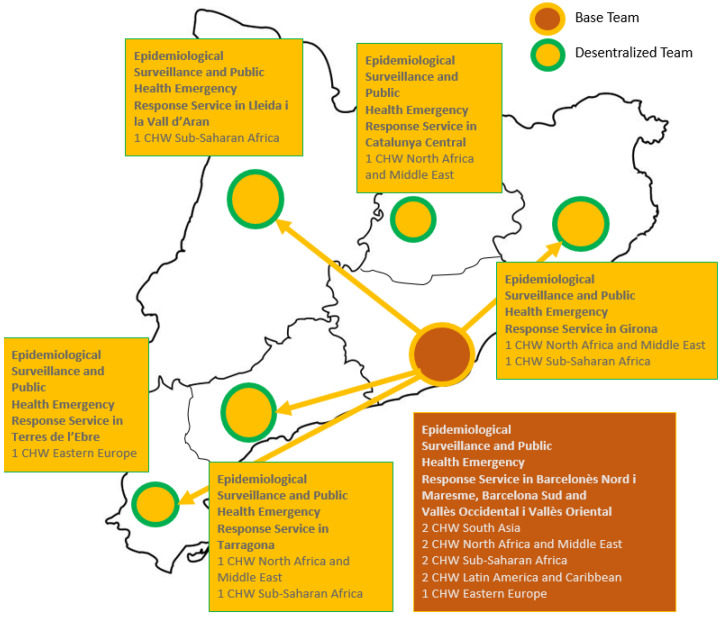
Distribution of CHW in Catalonia according to Epidemiological Surveillance and Public.

The distribution of the CHWs was made according to the needs detected in each territory. Seven women from different communities were recruited for the decentralized team. In addition, a group of five men and four women formed the Base Team and acted in their assigned territory but also could provide coordinated support to other territories to ensure project coverage. (Figure 1). All CHWs participated in the training session.

### 3.3. Implementation of HSUS

#### 3.3.1. Architecture and Data Privacy

Health SUrvey System, HSUS, is an information system based on a Client-Server architecture, which allows the collection and display of data associated with health surveys in real time. It incorporates a client-server solution to manage system’s data in a secure way, assuring its control and traceability once the data has entered the system.

The main advantage of the adopted design resides in the lack of sensitive data stored in the end user device, which forces the requirement of internet connection to be able to send and receive information from the backend server. For simplicity, we will not go into technical details of the implemented securitization, but overall it is done using certificates and cryptographic tokens to ensure confidentiality and avoid eavesdropping while preventing unauthorized users from accessing the system. In addition, sensitive data is stored in an encrypted database, which can only be accessed through a private API that cannot be directly reached from the internet.

**Figure 2:**
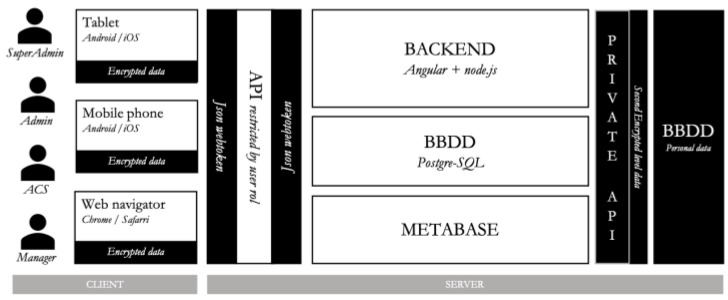
Component diagram of the HSUS architecture.

Figure 2 displays the different components of the HSUS system.

#### 3.3.2. System Functionalities

HSUS main objective is to collect, store and analyze data associated with health studies. However, it has other capabilities aimed at supporting CHW day-to-day workflow in order to help them to successfully organize and carry on their studies:

- User management: The system integrates role management module with a set of predefined roles, which limits the user scope.
- Plan and design community actions: The system incorporates a community actions management module to plan and design which actions will be performed as well as which promotion and/or epidemiological service units will be involved in its development.
- Request for new community actions: The platform lets promotion and/or epidemiological surveillance services request for new interventions.
- Real-time information analysis: The system integrates an open source tool, which helps to analyze non-sensitive data by portraying it in multiple customizable dashboards, either to show the real-time evolution of a single community action or the overall results of a group of them.
- Custom survey editor: The system administrators can build their own health survey models by choosing among the existent question blocks or by creating new ones.

#### 3.3.3. Real-Time Information System

**Figure 3:**
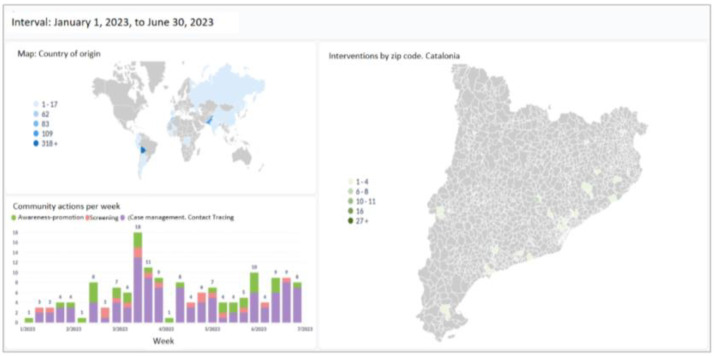
Example of geolocation community interventions in the Catalan territory, participants by country of origin and community interventions carried out by week.

In order to be able to analyse the non-sensitive data associated with a health study in real-time or once finished, a new analytic tool called Metabase was integrated into the system. Metabase lets the administrators define a set of customizable dashboards that can be defined directly through SQL queries or by means of a user-friendly interface that uses a drag and drop system to quickly extract the relevant information of the database’s tables. (Figure 3).

### 3.4. Community-Oriented Strategy

**Figure 4:**
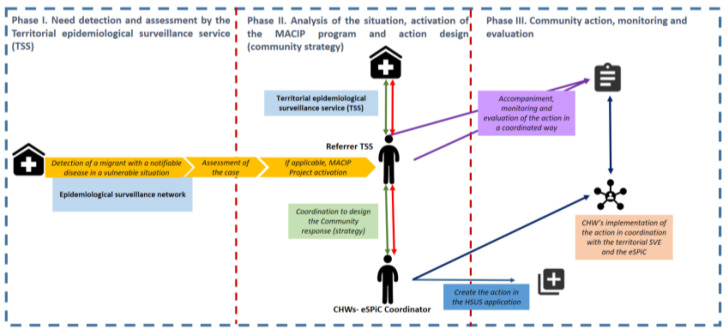
Structure, operating phases and methodology of the intervention.

The way the project operates is based on communication and coordination between the epidemiological surveillance network, the EsPIC and the group of ACWs, according to the different phases framed in Figure 4.

In order to determine the most appropriate community strategy according to the profile of the case to be intervened, the project comprises four approaches: community networking, culture-ally adapted health awareness and education, community screening, and community case and contact management.

Regarding the community networking, CHWs have identified the community assets of the communities and have established alliances with organizations and civil society.

**Figure 5:**
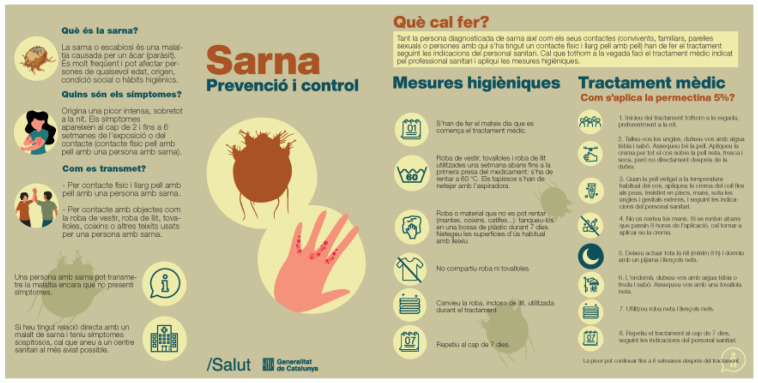
Scabies leaflet culturally adapted.

The second approach, based on culturally adapted health awareness and education has been addressed thought the the eSPiCTools. In this program, a new challenge in tackling scabies outbreaks has been identified. Accordingly, a process of co-creation of a new educational tool has been launched to empower people and improve their ability to respond to challenging health situations. The protocol for the prevention and control of scabies has been revised [23], along with health professionals and CHWs to identify needs and barriers to scabies prevention and control. This process has allowed the design of educational material to complement community actions taken by CHWs in addressing scabies outbreaks. So far, a leaflet has been designed and tested in a scabies outbreak with Pakistani community (Figure 5). Next step will be to validate this educational resource with other communities and contexts. In addition, audiovisual material will be developed to improve the cultural adaptability of the resource to different target communities.

This resource aims to facilitate the community case and contacts management strategy.

The third approach, community screening, has been used in the past mainly for CHD. The community-based in-situ screening for CHD and strongyloidiasis was carried out in an integrated approach for the Bolivian population.

The fourth and last approach, based on the community case management of notifiable diseases and contact tracing was realized through phone calls, mediation, informal meetings, accompaniment to medical appointments, home visits, and health awareness and education workshops, among others.

### 3.5. Project Evaluation

From 1 January to 30 June 2023, 117 community-based interventions have been implemented, reaching 677 people. 314 (46.4%) of the participants were women. The median age was 37 years (P25 27–P75 47) and the most represented population group was 25 to 44 years old (52.7%).

The community with the highest participation was Bolivian community (50.3% of the participants), followed by Pakistani community (16.6%), Bangladeshi (12.3%) and Moroccan (6.8%). 65.0% of the total population had secondary studies, and 46.3% had a stable employment situation. 49.8% had been resident in Catalonia for more than five years (Table 1).

Three different community-based strategies have been implemented: 73 community case and contacts management interventions, 17 community in-situ screenings (reaching 247 people) and 27 culturally adapted health awareness and education actions (reaching 358 people). The following infectious diseases have been addressed: tuberculosis, CHD, hepatitis C, typhoid, scabies, hepatitis B, mumps and tinea capitis (Table 2).

The population reached by each strategy is highly variable. 

The community in-situ screening strategy was used for CHD in the Bolivian community and for hepatitis C in the Pakistani community, as these are population groups with a higher prevalence of these diseases respectively. 

In contrast, for the community case and contacts management strategy, the program reached communities from Pakistan (28.9%), Morocco (22.4%), India (10.5%), Mali (6.6%), Senegal (6.6%) and other communities (13.2%), according to vulnerable cases with a communicable disease requiring the involvement of a CHW.

Finally, with the awareness-raising strategy, the communities with the highest number of participants were the Bolivian (39.1%), Bangladeshi (23.2%) and Pakistani (14.2%), as this strategy is complementary to the two previously mentioned, especially to improve participation in CHD and hepatitis C screening.

Vulnerability factors of participants were very different according to their region of origin. It is noted that more than half of the women participating in the MACIP project were from the Latin America and Caribbean region (62.3%).

Overall, the model reached 214 new arrivals (i.e., less than three years since its arrival in Europe), 353 people who reported being unemployed or in unstable employment, 262 people in an irregular administrative situation (i.e., not holding an identity document issued by Spanish authorities) and 47 people who did not have a health card. The level of education varied greatly for the different regions of origin, but it is notable that the Latin America and Caribbean region presented a higher level of education compared to the others (Table 3).

## 4. Discussion

In Catalonia, a community-based public model for the prevention and control of communicable diseases in migrant communities has been implemented. The program pursued a comprehensive and equitable approach: comprehensive because it considers health from all the spheres that conform it, i.e., also addressing the social determinants of health from an intersectional perspective. Equity because the interventions are carried out from a contextualized view of the community, focusing on the gap of health inequalities.

A strength of this model is that it allows reaching the undocumented migrant population. This group of population often does not access primary care or make less use of healthcare services, although they tend to have a higher incidence of infectious diseases of poverty [5], including NTDs. This model may contribute to facilitating the access of these communities to the health system, to reduce underdiagnoses and undertreatment of NTDs and other infectious diseases.

The integration of CHWs into epidemiological surveillance services has facilitated the identification and prioritization of needs that are being adapted according to the actual needs of the community. To date, the program has managed to tackle different infectious diseases throughout Catalonia, helping to reduce territorial inequality.

Recruitment and selecting the most appropriate person for the CHW role is one of the most essential elements contributing to a successful community health strategy. This is key to ensuring retention of CHWs, sustainability and acceptability, and successful outcomes of CHWs programs [14]. In addition, an active involvement of the community being targeted in the recruitment of CHWs enables that the CHW is trusted and accepted by the community [24]. In our program, gender equity, geographical location and experience in developing community health strategies were also highly considered.

Internationally, CHWs as individuals from minority communities are used to reach out to others in the community, as well as to provide direct services such as health education. By being known and respected by the community, they are key figures in linking the community to the health system. Moreover, they can engage people who have not previously sought care, establish cultural links, overcome distrust and contribute to doctor-patient communication, increasing the likelihood of patient follow-up and providing cost-effective health services to underserved communities [25].

The training process for CHWs should be adapted to the specific activities they are expected to perform and the context in which they will be working on the basis of local needs [14,24]. Furthermore, access to training has been described as an important factor in CHWs retention [14]. In this sense, the continuous training and active participation of CHWs in the training process aims to strengthen the program, integrating the perspective of CHWs and adapting the training needs according to the programs requirements.

Data collection by CHWs is a potentially significant but under-researched pathway to improving community health services [14]. The design of an IT tool that contributes to the implementation of a surveillance and epidemiological intelligence model with real-time information can be essential for program monitoring and evaluation. It also allows to see epidemiological trends or to know which community intervention strategy is the most appropriate for a particular community or population group.

Community-oriented actions are not implemented alone, but are accompanied by other interventions that ensure a holistic approach to community action. To decide on the most appropriate strategy to implement for each context, a literature search was conducted. The design and implementation of the strategy was discussed and adopted by both the epidemiological surveillance services and the eSPiC.

This program reaches out different communities through different community-based strategies that address multiple infectious diseases. For example, for tackling CHD, the focus is on reaching Bolivian communities, as they account for 53.9% of CHD estimated cases in Spain [26]. For this, health awareness and community in-situ screening has been implemented based on the successful results found in the literature for the management of this disease in this community [15].

The multi-focal approach of the project succeeds in reaching women, especially through the community-based screening strategy. The interaction of gender and migration status generates specific forms of vulnerability and determines the experiences, frequency and perception of health. Inequalities of power, sexual division of work and gender socialization—vectors of gender inequalities in health—are re-articulated through migration. As a result, migrant women have higher morbidity, poorer perceived health and more rapid loss of quality of life in the host country than men. Because of that, previous studies highlight the need to include a gender perspective in community interventions aimed at reducing vulnerability [27]. To this end, community-based screening strategy sought to be a mechanism for linking women to the health system. In a community-based intervention for the detection of CHD in Barcelona, it was observed that 71.2% of screening participants were women [16]. Another study found that the percentage of women participating in different screening strategies for CHD ranged from 54.7% to 81.5% [15]. These studies suggest that women show more concern and interest in screening, mainly because of feelings of guilt, worry and responsibility for possible congenital transmission [15,28]. Therefore, this community-based strategy can be key element in promoting gender equity within the programme.

Health awareness and education strategy culturally adapted have been shown in previous studies to be essential in improving access to diagnosis and treatment of hepatitis C [22] and CHD [15,16,17] in vulnerable populations in our context. These studies also highlight the role of community-based interventions involving CHWs in improving the acceptability and effectiveness of the strategy. In addition, this strategy contributes to promoting permanent behavioral change.

For case management and contact tracing, previous community health actions with CHWs were effective to localize lost children born from pregnant women with CHD and their families [27] or to improve contact tracing and treatment compliance among migrant communities [21,29]. In our programme, we have also seen that the role of CHWs may also be very beneficial in the prevention and control of other infectious diseases such as scabies in vulnerable groups.

This project is constantly being evaluated to adapt it to emerging needs. In this sense, it has been identified the need to incorporate the role of a CHW for the Chinese community, which represents 5% of foreign population in Catalonia in 2022 [30].

## 5. Conclusions

The implementation of a community-based model may be key to improving surveillance of NTDs and other communicable diseases, and thereby have a positive impact on the implications they have on Public Health. This model can contribute to reducing social inequalities in health, where the involvement of CHWs is essential to reaching migrant vulnerable communities, as they play a crucial role in addressing barriers related to access to health services.

Intervention strategies in migrant communities should be adapted to the specific characteristics of each group or individual. A community intervention involving both CHWs and epidemiological surveillance services may be more effective than classical communicable disease prevention and control activities, as the multidisciplinary nature of the team allows the inclusion of the perspective of both public health professionals and the community. In addition, they can build mutual trust with communities that may prove useful in the future for the development of strategies for health promotion and disease prevention and control.

Finally, one of the most important contributions that this model has, compared to other similar programs using CHW from migrant communities is the inclusion of the data collection system, which is able to use the network of CHW and community-based associations as epidemiological surveillance platform of population frequently missed or underreported in routine surveillance systems.

## Figures and Tables

**Table 1:** Socio-demographic characteristics of participants.

	Total	Community In-Situ Screening	Health Awareness and Education	Community Case and Contacts Management
N (%)	N = 677	N = 247 (36.5%)	N = 354 (52.3%)	N = 76 (11.2%)
Gender				
Female	314 (46.4%)	152 (61.5%)	131 (37.0%)	31 (40.8%)
Male	363 (53.6%)	95 (38.5%)	223 (63.0%)	45 (59.2%)
Age group				
0 to 24 years old	122 (18.2%)	35 (14.2%)	65 (18.6%)	22 (29.3%)
25 to 44	353 (52.7%)	120 (48.8%)	201 (57.6%)	32 (42.7%)
45 to 64	177 (26.4%)	85 (34.6%)	74 (21.2%)	18 (24.0%)
65 or more	18 (2.7%)	6 (2.4%)	9 (2.6%)	3 (4.0%)
Countries of origin				
Bolivia	340 (50.3%)	199 (80.6%)	138 (39.1%)	3 (3.9%)
Pakistan	112 (16.6%)	40 (16.2%)	50 (14.2%)	22 (28.9%)
Bangladesh	83 (12.3%)	-	82 (23.2%)	1 (1.3%)
Morocco	46 (6.8%)	-	29 (8.2%)	17 (22.4%)
India	17 (2.5%)	-	9 (2.5%)	8 (10.5%)
Colombia	14 (2.1%)	-	13.0 (3.7%)	1 (1.3%)
Mali	13 (1.9%)	-	8 (2.3%)	5 (6.6%)
Peru	10 (1.5%)	1 (0.4%)	5 (1.4%)	4 (5.3%)
Senegal	8 (1.2%)	-	3 (0.8%)	5 (6.6%)
Others	33 (4.9%)	7 (2.8%)	16 (4.5%)	10 (13.2%)
Years since arrival in Europe				
Less than 3 years	214 (33.0%)	35 (14.3%)	156 (45.2%)	23 (39.7%)
Between 3 and 5 years	111 (17.1%)	27 (11.0%)	74 (21.4%)	10 (17.2%)
More than 5 years	323 (49.8%)	183 (74.7%)	115 (33.3%)	25 (43.1%)

**Table 2:** Community-based strategies implemented for infectious diseases.

	Community Case and Contacts Management	Community In-Situ Screening	Health Awareness and Education
Chagas Disease		10	17
Hepatitis B	1		
Hepatitis C		7	1
Mumps	1		
Scabies	2		
*Tinea Capitis*	1		
Tuberculosis	65		9
Typhoid	3		
Total	73	17	27

**Table 3:** Vulnerability factors of participants by region of origin.

	Total	South Asia	North Africa and Middle East	Sub-Saharan Africa	Latin America and Caribbean	Eastern Europe
N (%)	N = 677	218 (32.3%)	47 (7.0%)	31 (4.6%)	377 (55.9%)	2 (0.3%)
Gender						
Women	314 (46.4%)	55 (25.2%)	22 (46.8%)	2 (6.5%)	235 (62.3%)	-
Years since arrival in Europe						
Less than 3 years	214 (33.0%)	117 (55.2%)	20 (50.0%)	21 (80.8%)	54 (14.7%)	1 (50.0%)
Between 3 and 5 years	111 (17.1%)	50 (23.6%)	8 (20.0%)	-	53 (14.4%)	-
More than 5 years	323 (49.8%)	45 (21.2%)	12 (30.0%)	5 (16.1%)	260 (70.8%)	1 (50.0%)
Economic situation						
Stable employment	304 (46.3%)	51 (24.2%)	8 (19.5%)	-	245 (65.9%)	-
Unstable employment	80 (12.2%)	51 (24.2%)	10 (21.3%)	6 (19.4%)	12 (3.2%)	1 (50.0%)
Unemployed	273 (41.6%)	109 (51.7%)	25 (56.1%)	24 (77.4%)	115 (30.9%)	1 (50.0%)
Educational level						
Without studies	62 (9.5%)	44 (21.0%)	6 (14.6%)	8 (27.6%)	4 (1.1%)	-
Primary studies	164 (25.0%)	90 (42.9%)	24 (58.5%)	14 (48.3%)	34 (9.1%)	1 (50.0%)
Secondary studies	429 (65.5%)	76 (36.2%)	11 (26.8%)	8 (27.6%)	334 (89.8%)	1 (50.5%)
Administrative situation						
Identity document (issued by Spanish authorities)	400 (60.4%)	78 (36.6%)	22 (48.9%)	10 (34.5%)	290 (76.9%)	-
Passport (in case of lacking a Spanish identity document)	262 (39.6%)	135 (63.4%)	23 (51.1%)	19 (65.5%)	82 (21.8%)	2 (100.0%)
Public healthcare registration card						
Yes	630 (93.1%)	207 (95.0%)	40 (85.1%)	22 (71.0%)	357 (94.7%)	2 (100.0%)
No	47 (6.9%)	11 (5.0%)	7 (14.9%)	9 (29.0%)	20 (5.3%)	-

## Data Availability

The data presented in this study are available on request from the corresponding author. The data are not publicly available due to privacy restrictions.
